# Routes of non-traditional entry into buprenorphine treatment programs

**DOI:** 10.1186/s13011-020-0252-z

**Published:** 2020-01-20

**Authors:** Tiffany Champagne-Langabeer, Michael W. Swank, James R. Langabeer

**Affiliations:** 1 School of Biomedical Informatics, University of Texas Health Science Center, Houston, TX USA; 2 McGovern Medical School, University of Texas Health Science Center, 7000 Fannin Street, Suite 600, Houston, TX 77030 USA

**Keywords:** Opioid use disorder, Buprenorphine, Treatment, Addiction

## Abstract

**Background:**

Excessive prescribing, increased potency of opioids, and increased availability of illicit heroin and synthetic analogs such as fentanyl has resulted in an increase of overdose fatalities. Medications for opioid use disorder (MOUD) significantly reduces the risk of overdose when compared with no treatment. Although the use of buprenorphine as an agonist treatment for opioid use disorder (OUD) is growing significantly, barriers remain which can prevent or delay treatment. In this study we examine non-traditional routes which could facilitate entry into buprenorphine treatment programs.

**Methods:**

Relevant, original research publications addressing entry into buprenorphine treatment published during the years 1989–2019 were identified through PubMed, PsychInfo, PsychArticles, and Medline databases. We operationalized key terms based on three non-traditional paths: persons that entered treatment via the criminal justice system, following emergencies, and through community outreach.

**Results:**

Of 462 screened articles, twenty studies met the inclusion criteria for full review. Most studies were from the last several years, and most (65%) were from the Northeastern region of the United States. Twelve (60%) were studies suggesting that the criminal justice system could be a potentially viable entry route, both pre-release or post-incarceration. The emergency department was also found to be a cost-effective and viable route for screening and identifying individuals with OUD and linking them to buprenorphine treatment. Fewer studies have documented community outreach initiatives involving buprenorphine. Most studies were small sample size (mean = < 200) and 40% were randomized trials.

**Conclusions:**

Despite research suggesting that increasing the number of Drug Addiction Treatment Act (DATA) waived physicians who prescribe buprenorphine would help with the opioid treatment gap, little research has been conducted on routes to increase utilization of treatment. In this study, we found evidence that engaging individuals through criminal justice, emergency departments, and community outreach can serve as non-traditional treatment entry points for certain populations. Alternative routes could engage a greater number of people to initiate MOUD treatment.

## Background

The continuous growth in the United States opioid epidemic has resulted in a significant number of lives lost and families destroyed, creating a public health emergency. From the years 1999 to 2016, over 351,000 people died of opioid-related causes [1], and according to a recent US Surgeon General Report, the overall cost of the opioid crisis in 2015 was over $500 billion [2]. The current national estimate is that 2 million individuals live with opioid use disorder (OUD), and a significant majority of these historically have not received agonist treatment (or medication-assisted treatment), leaving a large treatment gap despite improvements during the last few years [3, 4].

There are three FDA-approved medications to treat OUD: the partial opioid agonist buprenorphine, agonist methadone, and extended release naltrexone, an opioid antagonist. A recent study found that opioid agonist treatment cuts the risk of overdose fatality risk in half compared to no treatment [5]. Other studies and reports concur that MOUD saves lives and prevents relapses and other negative consequences [6; 7]. The challenge now remains how to get those affected into medical and behavioural treatment programs [3, 6]. Traditional paths of entry require patients to initiate or self-present for treatment to a provider or clinic.

In a study analysing data from 2013 to 2018 in the US, 350,000 patients with OUD were being treated with methadone and 112,000 with buprenorphine [7], although the most recent national drug use survey suggests that buprenorphine has made significant gains in 2017 and 2018 [8]. Although expansion of methadone treatment facilities has occurred, full penetration may be limited due to geographical constraints; furthermore, many counties, especially rural ones, have no methadone treatment programs at all [9].

Buprenorphine, unlike methadone, does not need to be administered in a drug treatment facility; and studies have shown that both in-office and home induction are effective [10, 11]. Buprenorphine can also be combined with an antagonist naloxone to limit the recreational potential and street value, as well as potential diversion relative to methadone [11]. A recent study from the United Kingdom also found that buprenorphine was significantly safer than methadone and had a lower toxicity profile with less risk of accidental overdose and death [12].

The small number of patients receiving buprenorphine/naloxone (hereafter called buprenorphine) suggests that treatment opportunities could be greatly expanded [13]. Increasing capacity of qualified and willing prescribers is necessary, but increased access does not necessarily translate to increased use. To ensure patients utilize treatment, they must first find entry into a program or provider; and the traditional model may not be feasible for a number of reasons. For this research, we define a traditional model of care where a patient requests and initiates treatment at an outpatient or inpatient clinic for an illness or disease, symptom, or disease. In reality, there are a multitude of barriers which impeded this model when seeking care for OUD include knowledge (e.g. what is MOUD?), geography (e.g. where can I find help?), financial (e.g. can I afford treatment?), and stigma (i.e. discrimination based on perception). In this review, we chose instead to focus on how persons with OUD are brought into treatment through non-traditional routes. We were primarily interested in entry into buprenorphine treatment through an emergency route (e.g. an encounter with hospital emergency departments), the criminal justice system, or facilitated by community or public health outreach.

Persons with OUD who experience an overdose on injected opioids may end up in the emergency room [14–17]. This creates an opportunity for change and makes this one potential route of buprenorphine induction. Illicit opioid use places individuals at an increased risk of interaction with the criminal justice system, including incarceration [18]. However, studies show the availability of MOUD, including buprenorphine, is very low in criminal justice settings [19], and this represents a missed opportunity for treatment. Finally, public health outreach is one other possible means of engaging patients into treatment. Previous studies have shown outreach to be useful in engaging patients in methadone treatment [20]; however, very little has been published on its application to buprenorphine treatment induction [21]. While methadone has multiple barriers to increased utilization (e.g., geographic barriers and stringent dispensing guidelines), buprenorphine can be prescribed by any physician that has met the training and license requirements set forth by the Drug Enforcement Agency (DEA) Drug Addiction Treatment Act of 2000 **(**DATA 2000) and nurse practitioners and physician assistants who have met the training and licensure requirements set forth by the Comprehensive Addiction and Recovery Act of 2016 (CARA) [22].

Our research focuses on how patients with OUD initially obtained treatment with buprenorphine. In selecting this research, we were driven in part by a perceived deficit in studies that looked at strategies to link patients to buprenorphine treatment through less conventional routes. The aim of this study is to examine the literature on buprenorphine treatment entry resulting from non-traditional routes, including emergency, criminal justice, and outreach settings.

## Methods

We conducted a systematic search and narrative review of the literature over the last 30 years (1989–2019) to evaluate the landscape of existing research for non-traditional routes to buprenorphine. The search was performed between March–April 2019 by the first and second authors. Four databases were included in this review: PubMed, PsychInfo, PsychArticles, and Medline. Search terms included a combination of medical subject headings (MeSH) and key words for PubMed, and key words for the remaining databases. A complete list of terms is found in Table 1.

**Table 1:** Search strategy and search terms

Search Strategy				
PubMed Database				
opiate substitution treatment [Mesh] OR opioid-related disorders/drug therapy [Mesh] OR opioid-related disorders/ rehabilitation [Mesh]	AND	buprenorphine/ therapeutic use [MeSH] OR buprenorphine [MeSH]	AND	law enforcement [MeSH] OR criminal law [MeSH] OR incarceration OR prisons [MeSH] OR prisons OR prison OR emergency responders [MeSH] OR emergency treatment [MeSH] OR emergency medical services [MeSH] OR emergency service, hospital [MeSH] OR outreach OR induction
PsychInfo, PsychArticles, and Medline Databases				
opiate substitution treatment OR opioid-related disorders OR drug therapy OR opioid-related disorders OR rehabilitation	AND	buprenorphine OR buprenorphine therapy OR buprenorphine treatment	AND	law enforcement OR criminal law OR criminal justice OR incarceration OR prisons OR prison OR emergency responders OR emergency treatment OR emergency medical services OR emergency department OR emergency room OR outreach OR community programs OR induction

Criteria for initial inclusion were the following: original research report in a peer-reviewed journal that explicitly examined buprenorphine treatment initiated either through an emergency department or emergency hospitalization, within or associated with the criminal justice system, or through community outreach. Measured outcomes had to include effects of buprenorphine treatment on retention, opioid use, opioid positive urine toxicology, re-incarceration, or other health outcomes such as hospitalization, utilization of emergency facilities, or death. International studies were included if the report was in English and available for review. Papers were excluded if they: did not present original research; were reviews, commentaries, or editorials; were individual case reviews; were published in a language other than English; were published prior to 1989; did not report any outcome data; or did not address entry into buprenorphine treatment.

**Fig. 1:**
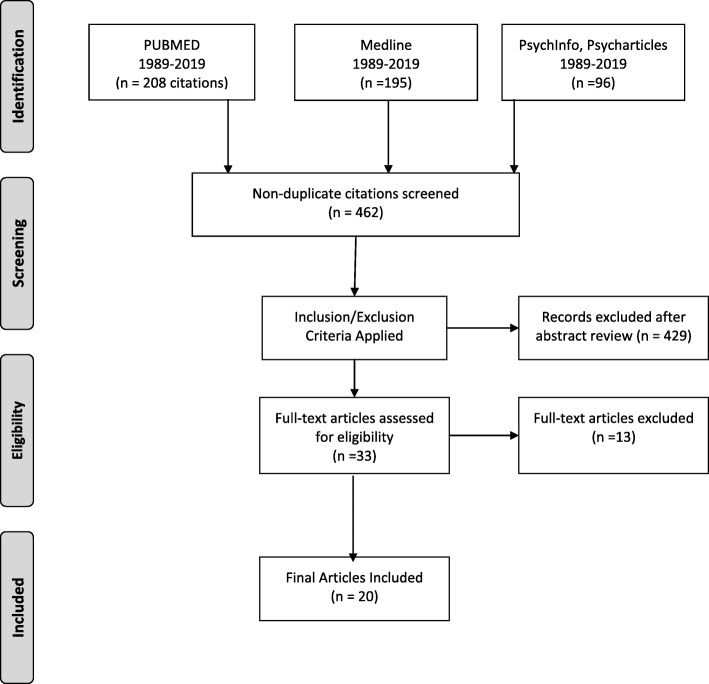
Systematic search and retrieval process

The initial search of 499 articles were indexed in an open access, web tool for review and abstraction [23]. After removing duplicates, a total of 462 citations remained. The first two authors performed a narrative review based on information obtained from titles and abstracts and applied inclusion and exclusion criteria, excluding 429 articles. Full-text articles were then examined further for inclusion/exclusion. An additional 13 articles were excluded because they did not report any outcome data (*n* = 9), or they were not relevant (*n* = 4), i.e., they did not address entry into buprenorphine treatment through an emergency route, criminal justice system, or facilitated by outreach. Twenty articles were selected for final inclusion and assessment. A schematic of the full search process is shown in Fig. 1.

## Results

We identified 20 articles with non-traditional routes of entry into buprenorphine. We found that the northeast region of the U.S. had the highest number of studies. Only one study from was from the South (Alabama), leaving the West and Midwest unrepresented in this cross-section of studies. Eight (40%) were RCT study designs, 35% retrospective analysis studies, and the remainder were pilot or cohort studies. The majority of the research was heavily concentrated towards the most recent years. All but 2 (90%) were small sample size studies, averaging less than 200 patients. Three articles focused on emergency settings; five were related to community outreach; and the majority (12) were related to criminal justice. Table 2 presents a summary of the studies included in this review.

**Table 2:** Summary of included study characteristics and findings

#	Article	Study Design	Sample	Route of Entry	Intervention	Results	Conclusion	Limitations
1	Gordon et al., 2018	RA	*N* = 199	CJ	Initiating buprenorphine treatment prior to versus after release from prison.	No significant differences.	Treatment condition did not predict likelihood of arrest.	Number of rearrests may have been biased. During the following 12 months after release, many remain detained.
2	Busch et al., 2017	RCT	*N* = 329	ED	Cost-effectiveness of ED-initiated buprenorphine.	Patient costs significantly lower in ED-initiated treatment group.	ED-initiated buprenorphine treatment is cost-effective.	Comparability of data. Length of follow-up was 30-days post-randomization.
3	Lee et al., 2017	RCT	*N* = 72	O	Predictors of retention in office-based treatment after hospitalization.	Prior treatment, older age, and non-minority status were associated with more time in office-based opioid treatment.	Linking hospitalized patients to office treatment may improve addiction treatment.	Small sample size; no measures of mental disorders other than PTSD.
4	Gordon et al., 2017	RCT	*N* = 211	CJ	Initiating buprenorphine treatment prior to versus after release from prison.	In-prison group had higher number of treatment days after release than those who without treatment in prison.	In-prison buprenorphine was correlated with more days of treatment after release.	Fewer women and mostly African American population; results may not be generalizable.
5	Riggins et al., 2017	Cohort	*N* = 305	CJ	Buprenorphine treatment retention among HIV-positive patients with a history of incarceration.	No significant differences in groups	Recently incarcerated were more likely to be homeless, unemployed, and previously diagnosed with mental illness.	As an observational study, clear causative relationships could not be established.
6	Finlay et al., 2016	RA	N = 48,689	CJ	Likelihood of US Veterans to receive treatment for opioid use disorder at Veteran Health Association hospitals.	Veterans exiting prison receive lowest rates of treatment among all justice-involved US Veterans.	Targeted efforts to reach prison-involved veterans necessary as they have lowest odds of receipt.	Study limited to veterans who received treatment at VHA facilities.
7	Sigmon et al., 2015	Pilot study	*N* = 10	O	Feasibility of interim buprenorphine treatment to bridge delays during patient navigation.	Opioid abstinence:70% of participants retained through 12-week treatment program.	Interim treatment might reduce illicit drug use and drug-related risk behaviors among waitlisted.	Unrandomized pilot trial with limited sample size.
8	D’Onofrio 2015	RCT	N = 329	ED	Determine success of three intervention options for ED patients with OUD.	After 30 days, group receiving buprenorphine reported greatest reduction of illicit opioid use per week.	ED-initiated buprenorphine vs. brief interventions and referral significantly increased engagement.	Study involved only physicians approved to prescribe buprenorphine,. May not be reflective most ED physicians.
9	Liebschutz et al., 2014	RCT	*N* = 139	O	Methods of treatment among hospitalized patients post-discharge.	Linkage (intervention) more likely to enter treatment in office setting than those in detox group (72% vs. 11.9%).	Initiation to treatment is effective for hospitalized patients not initially seeking addiction treatment.	Study conducted as single institution with an associated buprenorphine outpatient treatment program.
10	Gordon et al., 2014	RCT	N = 211	CJ	Success of buprenorphine treatment to addicted prison inmates nearing release versus after release	In-prison treatment group more likely to continue treatment post-release; women more likely to complete prison treatment than men (86% vs 53%)	Buprenorphine appears feasible and acceptable to inmates who are NOT opioid-tolerant	Study not generalizable to all geographic locations; 70% of participants were male.
11	Zaller et al., 2013	Pilot study	N = 44	CJ	Initiating treatment prior to release from incarceration and linking participants to community treatment.	Eleven of 32 participants remained in treatment for entire 6 months.	Initiating buprenorphine treatment during incarceration; continuing in community is feasible; may increase retention post-release.	Small sample size; self-report nature of data, particularly drug use and criminal history.
12	Schwarz et al., 2012	RA	*N* = 209	O	Effect of treatment retention on reducing ED utilization among treatment seeking patients.	Treatment retention was strongly correlated with a decline in ED visits (1 month = 1.6% decline per person).	Buprenorphine maintenance treatment significantly reduces ED utilization.	Lack of randomization does not allow for control of selection.
13	Lee et al., 2012	Cohort	*N* = 142	CJ	Comparing treatment retention and opioid misuse among those seeking treatment after release from jail.	Treatment retention over time was similar between groups.	Primary care appears to a feasible model of opioid treatment once released from incarceration.	Study participants were largely uninsured but received treatment through the study; whereas uninsured community referrals had no assistance.
14	Cropsey et al., 2011	RCT	*N* = 36	CJ	Efficacy of buprenorphine for relapse prevention among women in criminal justice system transitioning to community.	Treatment was effective in maintaining abstinence compared to placebo (92% placebo vs 33% buprenorphine were opioid positive per urinalysis).	Initiating buprenorphine in prison prior to release appears to reduce opioid use when participants reenter community.	Small sample size; limited generality as participants were women with criminal justice involvement.
15	Wang et al., 2010	RA	*N* = 166	CJ	Determine whether history of incarceration affects response to primary care office-based treatment.	Participants with history of incarceration have similar treatment outcomes with primary care office-based treatment than those w/o history of incarceration	Formerly incarcerated patients ar emore likely to have been treated with methadone, but do not have substantially different outcomes than those without prior incarceration.	Measurement of incarceration was self-reported and time incarcerated was grouped (patients with one month and multi-years were in same group).
16	Marzo et al., 2009	Cohort	N = 507	CJ	Describe the profile of imprisoned French opioid-dependent patients	77% of pts. received MAT at imprisonment, these patients were in poorer health & were more isolated than other population; 238/478 pts. were re-incarcerated within 3 years	MAT has increased in the criminal justice system in France, but maintenance therapy not associated with lower rate of reincarceration.	Conclusions on mortality are not well-supported as study was not designed for mortality analysis; pt. selection not random
17	Magura et al., 2009	RCT	*N* = 116	CJ	Test the efficacy of buprenorohine versus methadone while incarcerated and follow-up.	Patients in buprenorphone group reported to treatment significantly more than patients taking methadone.	There were no significant differences between groups for re-incarceration, relapse, re-arrests.	Findings may not be generalizable in other nations where methadone distribution protocols vary.
18	D’Onofrio et al., 2017	RA	*N* = 290	ED	Outcomes assessment of previous RCTs to determine long-term outcomes.	Patiengts in the buprenorphine group showed greater engagement in treatement at 2 months which was statistically significant.	Gains did not persist after 2 months when measure at the 6 and 12 month time points.	Buprenorphine treatment initiatied in the ED was associated with increased engagement during 2 month interval when treatment was continued at PCP.
19	Vocci et al., 2015	RA	*N* = 104	CJ	Assessed prior RCT to examine if induction into buprenorphine during incareceration was associated with seeking treatment post-release.	Participants were rapidly inducted onto buprenorphine with no serious side effects whle incarecerated.	Buprenorphine administered to non-opioid tolerant adults may be used to reduce rates of withdrawal and re-use post-incarceration.	None noted.
20	Cushman et al., 2016	RA	*N* = 113	O	To assess whether inpatient initiation to buprenorphine and linkage to counselling reduces illicit opioid use.	Patients who were linked to outpatients ervices versus patients in detox (inpatient) were more successful in the short term.	Differences did not persist between groups (linking versus detox) as far as injection opiate use at 1, 3, or 6 month timepoints.	May not be generalizable with a small population.

Abbreviations: *RA* Retrospective Analysis, *RCT* Randomized Controlled Trial, *CJ* Criminal justice system, *ED* Emergency department, *O* Outreach

### Entry into treatment from the emergency department

D’Onofrio et al. (2015) examined emergency department (ED)-initiated buprenorphine treatment in a randomized controlled trial (RCT) with 329 opioid dependent patients [24]. After screening, patients were randomized to intervention groups. The primary outcome measure was engagement and retention in treatment at 30 days; secondary outcomes included number of days of illicit drug use and use of addiction treatment services. The main findings from this study concluded that ED-initiated buprenorphine treatment significantly increased engagement in addiction treatment and decreased use of inpatient addiction treatment services. The authors concluded that although this study supports the use of ED-initiated buprenorphine, replication in other settings would be required before widespread implementation. A secondary analysis of this study looked at long-term outcomes at 2, 6, and 12 months following ED-initiated buprenorphine [25]. Patients were transitioned to outpatient treatment settings where they received buprenorphine (or tapered off the medication) and were followed over a 12-month period. Measured outcomes included treatment engagement, illicit drug use, HIV risk, and urine toxicology. Although the buprenorphine group had greater engagement and lower self-reported illicit drug use at 2 months, these differences did not persist at the 6- and 12-month time points [25].

Busch and colleagues (2017) examined the cost-effectiveness of ED-initiated buprenorphine in a subset of patients (*n* = 244) after completion of a 30-day assessment [26]. Cost measures included utilization of healthcare and treatment resources, labor costs of medical practitioners involved in treatment, drug costs, costs associated with crime, and patient time costs. They also concluded that overall ED-initiated buprenorphine was a cost-effective treatment option. The conclusion here was that ED-initiated buprenorphine is a cost-effective and useful route to engagement in MOUD, but additional work remains to determine how to best retain patients in long-term treatment with the goal of reducing or eliminating illicit opioid use.

### Seeking patients through outreach

In a randomized controlled trial from Liebschutz et al. (2014), opioid-dependent patients hospitalized for reasons other than opioid dependence were recruited from an inpatient facility [27]. The study compared a 5-day buprenorphine taper with buprenorphine induction and transition to maintenance therapy. Measured outcomes were entry into maintenance treatment, retention at 1, 3, and 6 months, and self-reported prior 30-day use of illicit opioids. Buprenorphine linkage was associated with more favourable outcomes than detoxing alone in terms of entry (72.2 vs 11.9%), 6-month retention (16.7 vs 3.0%), and illicit opioid use. A secondary analysis looking at illicit opioid use at 1, 3, and 6 months found no differences [28]. An additional brief report looked at predictors of treatment engagement and found previous buprenorphine treatment, more days hospitalized, and higher symptoms of post-traumatic stress disorder (PTSD) predicted higher number of days in office-based buprenorphine therapy [29]. A study which recruited patients through community fliers found patients inducted into buprenorphine treatment during the first week of treatment with bi-weekly visits up to 12 weeks showed better outcomes than wait-listing on all measures, and retention at 12 weeks was 70% [30]. Patients rated the program highly and money spent on drugs decreased.

The final outreach study looked at how buprenorphine treatment affects ED use and hospitalizations [31]. Patients were recruited from the New Haven, CT Community Healthcare Van (CHCV), which is linked to the syringe exchange program and is the first mobile induction and maintenance program in the US. They found that buprenorphine treatment reduced ED utilization but had no effect on number of hospitalizations or length of stay.

### Criminal justice-associated entry into buprenorphine treatment

Criminal justice-associated entry into buprenorphine treatment represents a majority of the articles. The studies presented here can be roughly conceptualized as three types with a main focus on: 1) offering buprenorphine treatment either pre- or post-release from incarceration; 2) incarceration status on buprenorphine treatment outcomes; and 3) effect of buprenorphine treatment on incarceration status or criminality.

#### Pre- vs post-release buprenorphine

A randomized controlled trial [32] and two secondary analyses [32, 33] used a 2 × 2 × 2 factorial design to explore the effects of in-prison treatment: buprenorphine or counselling only, post-release service center (opioid treatment center or community health center), and gender. Individuals assigned to the buprenorphine treatment condition were more likely to enter treatment than those assigned to counselling only and were more likely to enter community treatment after release. A follow-up study at 12 months [33] measured the following outcomes: days of community treatment, days of heroin use, crime, positive urinalysis for opioids or cocaine, and gender effects. Patients who initiated buprenorphine in prison had a higher number of days of community treatment but did not differ from the counselling only group on any other measures.

An earlier pilot study [34] reported on the feasibility of initiating buprenorphine prior to release on a small group (*n* = 44) of prisoners. Initiation of buprenorphine pre-release resulted in faster engagement with a prescriber post-release, as well as much longer treatment duration (24 vs 9 weeks). A pilot RCT examining women on parole or probation and found that at 12 weeks, when treatment concluded, buprenorphine was much more effective than placebo in reducing positive opioid urine tests. However, at a 3-month follow-up, there was no longer any difference [35].

#### Incarceration status and buprenorphine effectiveness

The next group of articles examines how incarceration status and prior incarceration history can impact the efficacy of buprenorphine treatment. In an analysis of a randomized control trial, the authors found that prior incarceration had no effect on either treatment retention or illicit drug use as measured by urinalysis [36]. The authors concluded that despite major demographic differences between previously versus never incarcerated, the effectiveness of buprenorphine was the same in both groups; and prescribing physicians can treat this patient population without bias. Two additional studies found similar results. In one cohort study, they found no differences in treatment retention between previously incarcerated versus community-referred groups [37]. In another multisite cohort study, they found that recent incarceration was not associated with any differences in 6- or 12-month treatment retention or self-reported opioid use [38]. A large multi-site retrospective cohort study (*n* = 48,689) examined national Veterans Health Administration (VHA) clinical and pharmacy records of veterans diagnosed with opioid use disorder and found that veterans with criminal justice involvement had reduced odds of receiving MOUD compared to others [39].

#### Buprenorphine treatment and incarceration status

This group of articles examines how buprenorphine treatment impacts incarceration status and criminality. In this secondary analysis of a randomized clinical trial discussed earlier [35], the authors examined the effects of pre- and post-release buprenorphine initiation on arrest outcomes over a twelve-month period: rearrested, time to re-arrest, number of rearrests, and severity of charges [40]. The results found 43.1% were rearrested, but there was no effect of treatment condition on any arrest outcome measure. Similarly, a study that also included a methadone treatment group along with buprenorphine found that neither medication had any significant effects on re-arrest, severity of crime, or re-incarceration [41]. A larger prospective observational study of French prisoners (*n* = 507) looked at three-year outcomes and found that buprenorphine treatment was not associated with any change in the rate of re-incarceration [42]. Another study concluded that the risk of re-incarceration and mortality remains high and further prevention is needed to elevate the health of this population [43]. In addition, the focus on re-incarceration rates, rather than treatment retention in general, could be problematic with these studies.

## Discussion

We found modest evidence of programs that utilized non-traditional entry routes into buprenorphine treatment. Twenty studies published over this long time-frame is relatively low. The studies we did find were largely small pilot studies based in the northeast. Future research should focus on larger sample sizes, randomization, and broader geographic representation. Of the studies identified however, findings suggest that there is evidence that non-traditional routes can be used to engage patients with opioid use disorder into buprenorphine treatment. Greater access to non-traditional routes could improve awareness to treatment alternatives, which in turn could increase the number of patients who choose to engage in MOUD overall.

The hospital emergency department appears to present a significant opportunity to initiate treatment. Encouraging greater use of buprenorphine as standard of care in the ED could benefit a large number of patients. The studies identified suggest that buprenorphine in the ED could also be a cost-effective strategy, given its potential for greater availability and distribution [44, 45]. Yet most of these studies were conducted only out of a single city (New Haven), thus limiting generalizability across all regions and settings.

Outreach is another approach that can provide awareness and motivation for people who use drugs about available treatment options, as well as navigating them to treatment. We found only two articles that utilized community-based outreach: one using flyers distributed and posted throughout the community [29], and the other utilizing a mobile healthcare van linked to needle exchange [30]. An additional study [26] and its secondary analyses [27, 28], is considered outreach because patients, while hospitalized for other health problems, were actively engaged in buprenorphine treatment. Hospital-based outreach represents an ideal environment because not only can they be an effective site of screening, they can also initiate treatment without delay.

With regards to criminal justice, we found prisoners have a much higher rate of OUD and represent a great opportunity to increase access to treatment [46–48]. The studies we found looked at differences between pre- and post-release initiation of buprenorphine treatment. The premise is that initiating buprenorphine treatment prior to release may inoculate or protect patients upon release where they face a much higher risk of death during their first 2 weeks’ post-release. Providing prisoners greater opportunity to initiate MOUD treatment prior to release is a potential strategy.

There are policy considerations that could help increase access to buprenorphine treatment through non-traditional routes. Linkage to long-term maintenance treatment was mentioned by multiple studies, and identifying mechanisms to improve continuity of care beyond the initial induction of buprenorphine is worthy of additional research. Increasing access to buprenorphine through non-traditional routes is promising, but this still requires availability of treatment providers. While buprenorphine treatment has some distribution advantages over other medications, treatment gaps and barriers still exist. Removing these barriers, such as requiring a separate license waiver to treat and even the use of telemedicine for initial consultation, represents one opportunity to further increase access [49]. In addition, many of these specially licensed physicians are not actually treating OUD patients with buprenorphine, either because of stigma regarding OUD patients and treatment [50], financial factors [51], or other perceived negative factors [50, 52]. Those with waivers tend to be in metropolitan areas, leaving a larger gap in rural counties [53].

From a public health perspective, additional resources should focus on outreach mechanisms that can identify patients and more proactively engage them into treatment earlier, rather than waiting for them to present at emergency departments or treatment facilities. Involvement from first responders, such as police and emergency medical services, to targeted neighborhoods impacted the most by the opioid epidemic could help improve awareness of treatment across communities. Encouraging and incentivizing a larger proportion of hospital emergency departments to initiate buprenorphine treatment will require significant changes in pharmacy formularies, reimbursement, and provider training. Yet, this investment in the ED as an early access point represents a significant opportunity for increasing access.

This research is novel in that while most studies have focused solely on analyzing access to potential treatment, limited evidence exists on mechanisms to increase rates of entry into treatment. We conclude that alternative routes to buprenorphine could reduce treatment delays for persons with opioid use disorder.

There are limitations to this research. First, as with all reviews, there is a possibility we did not include all articles because of our search terms, the databases we chose to query, or our relatively tight inclusion criteria. We also chose not to include articles or reports that were not published in peer-reviewed academic journals, which could limit the findings. While many of the studies we included were randomized designs, sample size, statistics, and outcomes were all varied. Despite these limitations, the strategies that we have reviewed in this research appear to be effective at improving treatment entry through non-traditional routes. Further research should examine other paths to entry and attempt to compare long-term outcomes of these non-traditional routes against more traditional paths. The implications from a policy perspective from these findings should also be explored.

## Conclusion

Despite research suggesting that increasing the number of DEA-waivered physicians who prescribe buprenorphine would help with the opioid treatment gap, little research has been conducted on alternative routes to increase utilization of treatment. In this study, we found that identifying individuals through non-traditional routes—including criminal justice, emergency departments, and community outreach—can be used to engage a greater number of individuals to initiate MOUD treatment.

## Data Availability

All data generated or analysed during this study are included in this published article and its supplementary information files.

## Abbreviations

CJ: Criminal Justice
DATA 2000: Drug Addiction and Treatment Act of 2000
DEA: Drug Enforcement Agency
ED: Emergency Department
OUD: Opioid Use Disorder
RCT: Randomized Controlled Trial
SAMHSA: Substance Abuse and Mental Health Services Administration
US: United States
VHA: Veterans Health Administration
